# Characterization of an Environmental Multidrug-Resistant *Acinetobacter seifertii* and Comparative Genomic Analysis Reveals Co-occurrence of Antimicrobial Resistance and Metal Tolerance Determinants

**DOI:** 10.3389/fmicb.2019.02151

**Published:** 2019-09-18

**Authors:** João Pedro Rueda Furlan, Otávio Guilherme Gonçalves de Almeida, Elaine Cristina Pereira De Martinis, Eliana Guedes Stehling

**Affiliations:** Departamento de Análises Clínicas, Toxicológicas e Bromatológicas, Faculdade de Ciências Farmacêuticas de Ribeirão Preto, Universidade de São Paulo, Ribeirão Preto, Brazil

**Keywords:** *Acinetobacter seifertii*, multidrug-resistant, whole genome sequencing, resistome, virulome

## Abstract

*Acinetobacter calcoaceticus-Acinetobacter baumannii* complex is considered one of the main causes of hospital-acquired infections. *Acinetobacter seifertii* was recently characterized within this complex and it has been described as an emergent pathogen associated with bacteremia. The emergence of multidrug-resistant (MDR) bacteria, including *Acinetobacter* sp., is considered a global public health threat and an environmental problem because MDR bacteria have been spreading from several sources. Therefore, this study aimed to characterize an environmental MDR *A. seifertii* isolate (SAb133) using whole genome sequencing and a comparative genomic analysis was performed with *A. seifertii* strains recovered from various sources. The SAb133 isolate was obtained from soil of a corn crop field and presented high MICs for antimicrobials and metals. The comparative genomic analyses revealed ANI values higher than 95% of relatedness with other *A. seifertii* strains than *A. calcoaceticus-A. baumannii* complex. Resistome and virulome analyses were also performed and showed different antimicrobial resistance determinants and metal tolerance genes as well as virulence genes related to *A. baumannii* known virulence genes. In addition, genomic islands, IS elements, plasmids and prophage-related sequences were detected. Comparative genomic analysis showed that MDR *A. seifertii* SAb133 had a high amount of determinants related to antimicrobial resistance and tolerance to metals, besides the presence of virulence genes. To the best of our knowledge, this is the first report of a whole genome sequence of a MDR *A. seifertii* isolated from soil. Therefore, this study contributed to a better understanding of the genetic relationship among the few known *A. seifertii* strains worldwide distributed.

## Introduction

*Acinetobacter* spp. are non-fermenting Gram-negative bacilli (NFGNB) ubiquitous in the environment and considered one of the main causes of hospital-acquired infections. *Acinetobacter seifertii* was recently characterized as belonging to the *Acinetobacter calcoaceticus-Acinetobacter baumannii* complex, which also includes *A. calcoaceticus*, *A. baumannii*, *Acinetobacter nosocomialis*, and *Acinetobacter pittii* (Nemec et al., 2011, 2015). *A. seifertii* has been described as an emergent pathogen, being reported in different human infections, including bacteremia (Cayô et al., 2016; Kishii et al., 2016; Yang et al., 2016).

Multidrug-resistant (MDR) bacteria, including *Acinetobacter* sp., carrying antimicrobial resistance genes (ARGs) have been reported worldwide from different sources, such as soil and water (Hrenovic et al., 2017; Furlan et al., 2018; Higgins et al., 2018; Furlan and Stehling, 2019). Bacterial resistance to antimicrobials has been considered a global public health and an environmental problem since soil and water sources are described as potential reservoirs and disseminators of antimicrobial-resistant bacteria as well as their ARGs, which is worrying (Berendonk et al., 2015).

Besides that, the presence of metals in the environment can co-select antimicrobial-resistant bacteria since efflux systems are a common mechanism for bacterial resistance to heavy metals and to antimicrobials (Seiler and Berendonk, 2012; Wales and Davies, 2015). Due to the importance of emerging environmental pathogens, this study aimed to characterize an environmental MDR *A. seifertii* isolated from soil and compare it through the whole genome sequencing with previously described *A. seifertii* strains obtained from different sources (i.e., human, animal, and environment).

## Materials and Methods

### Bacterial Isolation

Soil samples were collected between 2015 and 2017 from several cities belonging to the five Brazilian regions. For each soil sample, 1 g was added in 5 mL of Luria-Bertani (LB) broth (Oxoid, United Kingdom) and incubated at 37°C for 24 h. Then, 100 μL were seeded on MacConkey Agar (Oxoid, United Kingdom) and incubated at 37°C for 24 h. Finally, the morphologically different colonies were selected and stocked in LB broth plus 15% glycerol at −80°C. A total of 150 isolates was obtained and identified by sequencing of 16S rDNA (Weisburg et al., 1991). All these isolates have been previously studied and one of them, SAb133 isolate, was identified as *A. seifertii* and used in this study.

### Antimicrobial Susceptibility Testing

Minimum inhibitory concentration (MIC) assay was used to determine the antimicrobial susceptibility profile according to Clinical and Laboratory Standards Institute (CLSI; M100, 27th ed). The antimicrobials tested were ampicillin-sulbactam, ceftriaxone, cefotaxime, ceftazidime cefepime, imipenem, meropenem, gentamicin, tobramycin, tetracycline, ciprofloxacin, levofloxacin, and trimethoprim-sulfamethoxazole. The pattern of antimicrobial resistance was determined according to Magiorakos et al. (2012).

### Metal Tolerance Profile

Metal tolerance profile was determined according to Deredjian et al. (2011) using Trypticase Soy Agar (TSA) diluted 10-fold plus 5, 10, 20, 50 mmol/L of zinc (Zn^2+^), 0.5, 1, 2, and 5 mmol/L of copper (Cu^2+^); 9.76 × 10^–3^, 0.1, 1, and 5 mmol/L of cobalt (Co^2+^); 10 and 50 μmol/L of mercury (Hg^2+^); 0.6, 1.25, and 2.5 mmol/L of cadmium (Cd^2+^); 0.25, 1.25, 6.25, 12.5, 25, 50, 62.5, and 125 mmol/L of selenite (SeO_3_)^2–^; 0.25, 1.25, 6.25, 12.5, 25, 50, 62.5, and 125 mmol/L of tellurite (TeO_3_)^2–^; 5, 10, 15, 20, 25, 30, 35, 40, 45, and 50 of arsenic (AsO_2_)^–^; 0.05, 0.1, 0.5, 1, 2.5, 5, 10, 15, 20, and 25 mmol/L of nickel (Ni^2+^); 0.05, 0.1, 1, 2, 5, 10, and 15 mmol/L of chromium (Cr^2+^); 1, 2,5, 5, 7.5, 10, 15, 30, 50, 75, and 100 mmol/L of magnesium (Mg^2+^). Metal tolerance was considered at the last concentration of bacterial growth.

### Whole Genome Sequencing (WGS)

Genomic DNA of SAb133 isolate was extracted using the PuriLink^TM^ Genomic DNA Mini Kit (Thermo Fisher Scientific, United States). Whole genome sequencing was performed using the Illumina MiSeq platform (Illumina Inc., EUA) with 250-bp paired-end reads and *de novo* assembly was performed using SPAdes v.3.9 (Bankevich et al., 2012). The SAb133 genome was annotated using NCBI prokaryotic genome annotation pipeline (PGAP) v.3.2 (Tatusova et al., 2016).

### Obtaining Genome Data

The full genome annotation of the 11 *A. seifertii* strains (Table 1) employed in this study for comparative analysis against the prospected *A. seifertii* SAb133 was downloaded from NCBI GenBank database on 12th August 2019 (Nemec et al., 2015; Roach et al., 2015; Yang et al., 2016; Cerezales et al., 2018). To add contrast in the comparative study were also downloaded the strains *A. baumannii* AB030 (GenBank accession no. CP009257), *A. pittii* ATCC 19004 (GenBank accession no. KB849785), *A. calcoaceticus* DSM 30006 (GenBank accession no. KB849778) and *A. nosocomialis* 28F (GenBank accession no. CBSD020000001) from the same database in the same date (Loewen et al., 2014).

**TABLE 1:** Data of *A. seifertii* genomes used in this study.

***A. seifertii* strains**	**Source**	**Sample**	**Country**	**Genome size (bp)**	**GC %**	**Genes**	**Proteins**	**Contigs**	**GenBank accession no.**
SAb133^a^	Environment	Soil	Brazil	3,882,472	38.6	3715	3566	30	SNSA00000000
KCJK7915	Environment	Water	United States	3,950,692	38.5	3776	3621	163	QAYP00000000
KCJK1723	Cattle	Feces	United States	3,884,778	38.5	3649	3508	120	LYQI00000000
1334_ABAU	Human	–	United States	4,143,123	38.6	4268	3735	681	JVTF00000000
MI421-133	Human	Catheter	Bolivia	4,039,753	38.5	3863	3718	222	PHFF00000000
MI30-324	Human	Abscesse secretion	Bolivia	4,051,078	38.4	3842	3700	113	PGPD00000000
V1371	Human	Knee-joint exudate	Bolivia	3,987,277	38.4	3932	3751	261	PHFG00000000
C917	Human	Blood	China	3,900,662	38.5	3739	3681	203	APCT00000000
A354	Human	Sputum	China	3,983,262	38.5	3713	3594	67	LFZQ00000000
A360	Human	Urine	China	3,948,160	38.5	3792	3632	137	LFZR00000000
A362	Human	–	China	4,344,373	38.5	4125	4009	114	LFZS00000000
NIPH973	Human	Ulcer	Denmark	4,212,819	38.6	4180	3890	26	APOO00000000

*^*a*^Data from this study.*

### Phylogenetic Analysis

The phylogenetic analyses were conducted based on the bioinformatics workflow performed by Turner et al. (2018). A maximum likelihood (ML) phylogenetic tree was drawn based on the full whole-genome sequences annotation available in GenBank to achieve a phylogenetic snapshot of the closed relationship among the *A. seifertii* strains. Firstly, were captured the single-copy orthologous genes in each strain, including *A. baumannii* AB030, *A. pittii* ATCC 19004, *A. calcoaceticus* DSM 30006, and *A. nosocomialis* 28F, which were added as out-groups to enhance the power of discrimination in the phylogenetic analysis. The single-copy orthologous genes were identified using the get_homologs pipeline (Contreras-Moreira and Vinuesa, 2013) and choosing as reference genome the first *A. seifertii* (NIPH973) identified.

The following parameters were selected in the command line options –M, –t, 16, –r NIPH973.gbff -e. The single-copy orthologous search was conducted with the OrthoMCL algorithm (Li et al., 2003) and the orthologous clusters were limited to single-copy orthologous genes present in all 16 genomes. The predicted orthologous clusters were aligned against each other using MUSCLE version 3.8.3 (Edgar, 2004) with default parameters through command line options. The generated alignments were trimmed using trimAl Beta version 1.4 (Capella-Gutiérrez et al., 2009). Next, the quality-processed alignments were concatenated in a one single FASTA file using UNIX command line.

The ML phylogenetic tree was built using the IQ-Tree software version 1.6.10 (Nguyen et al., 2015) based on the concatenated FASTA file as input. Thus, 1.000 ultra-fast bootstraps replicates (Minh et al., 2013) were performed and the best-fit model using the Bayesian Information Criterion (BIC – TIM2 + F + R10) was determined according to ModelFinder (Kalyaanamoorthy et al., 2017). The phylogenetic tree was visualized using FigTree software version 1.4.4. In regard for possible conflicting phylogenetic signals, a Neighbor-Net phylogenetic network (Bryant and Moulton, 2004) was drawn based on the previous concatenated sequences with SplitsTree software v.4.14.4 (Huson and Bryant, 2006) using default parameters and applying 1.000 bootstraps.

### Genome Relatedness and Similarity

The analysis of genetic relatedness among the *A. seifertii* strains and *A. baumannii* AB030, *A. pittii* ATCC 19004, *A. calcoaceticus* DSM 30006, and *A. nosocomialis* 28F was performed using the FastANI method that computes the relatedness (Average Nucleotide Identity – ANI) among all orthologous genes in whole-genome sequences, reflecting strains of the same species if they possess ANI ≥95% (Jain et al., 2018). Another way to compare closely related strains is based on the genomic similarity, and to address this aim, all 16 strains were compared through a visual plot of similarity ranging from 90, 96, and 100% using the blast ring image generator tool selecting *A. seifertii* NIPH973 as reference genome (Alikhan et al., 2011).

### Pangenome Analysis and Cloud Genome Determination

The pangenome structure was determined using the get_homologs tool with parameter *t* = 0 to obtain all orthologous clusters among the nine *A. seifertii* strains. The constituents of the *A. seifertii* pangenome were determined: core genome and accessory genomes (shell and cloud) using the script parse_pangenome_matrix.pl. To identify novel putative single-copy regions in these genomes, conceptually related to cloud genome, an accessory composition of the pangenome, the Panseq tool (Laing et al., 2010) was used selecting the “Novel Regions Analysis” pipeline with parameters for minimum region size of 500 bp and percent identity cut-off of 85%, and the *A. seifertii* NIPH973 was chosen as reference.

The resulting FASTA file containing the DNA sequences of presumptive novel genomic regions of each strain was analyzed on the GO-FEAT platform (Araujo et al., 2018). The output provided by GO-FEAT has comprised the gene ontology (GO) annotation that are categories divided in terms of Molecular Function, Cellular Component and Biological Process (Ashburner et al., 2000). The results regarding novel regions were summarized by biological processes according to GO annotation provided by the GO-FEAT platform.

### Whole Genome Sequencing Analysis

Genomic islands were detected using IslandViewer 4 associated with SIGI-HMM algorithm (Waack et al., 2006; Bertelli et al., 2017). Prophage-related sequences were identified using PHAge search tool enhanced release (PHASTER) (Zhou et al., 2011; Arndt et al., 2016). Insertion sequences (ISs) were searched using ISfinder (Siguier et al., 2006). Plasmids were identified using PlasmidFinder 2.0 (Carattoli et al., 2014) and *A. baumannii* PCR-based replicon typing (AB-PBRT) *in silico* method (Bertini et al., 2010). Antimicrobial resistance genes (ARGs) and chromosomal point mutations (CPMs), metal tolerance genes (MTGs), and *A. baumannii* known virulence genes (ABKVGs) were analyzed using Resfinder 3.1, BacMet database and Virulence Factors database, respectively (Chen et al., 2005; Zankari et al., 2012; Pal et al., 2014). Core genome multi-locus sequence type (cgMLST) was determined using BacWGSTdb (Ruan and Feng, 2016).

## Results

### Isolate, Resistance Profile to Antimicrobials and Tolerance Profile to Metals

The SAb133 isolate was obtained in 2016 from a soil sample cultivated with corn in Ribeirão Preto City, São Paulo State, Brazil (GPS 21.200133S and 47.872724W). This isolate presented high MICs, being resistant to ampicillin-sulbactam (64 μg/mL), ceftazidime (32 μg/mL), cefotaxime (>256 μg/mL), ceftriaxone (>256 μg/mL), tetracycline (>256 μg/mL), gentamicin (32 μg/mL), and tobramycin (32 μg/mL). The SAb133 isolate was classified as MDR since it presented resistance to ≥1 antimicrobial in ≥3 categories. The SAb133 isolate presented different metal tolerance profiles, including magnesium (>100 mmol/L), arsenic (30 mmol/L), tellurite (6.25 mmol/L), selenite (6.25 mmol/L), copper (5 mmol/L), zinc (5 mmol/L), chromium (2 mmol/L), cobalt (1 mmol/L), nickel (1 mmol/L), and cadmium (0.6 mmol/L).

### Genome Sequencing

The draft genome of SAb133 isolate was comprised of 30 contigs totaling of 3,884.033 (2 × 250-bp) paired-end reads reached by a 244× sequencing coverage. A total of 3,566 protein-coding sequences, 69 pseudogenes, 64 tRNAs, 12 rRNAs, and 4 ncRNAs were identified, with GC content of 38.5%. This Whole Genome Shotgun project was deposited at DDBJ/ENA/GenBank under the accession SNSA00000000. The version described in this paper is version SNSA01000000 (Table 1).

### Phylogenetic Analyses

**FIGURE 1:**
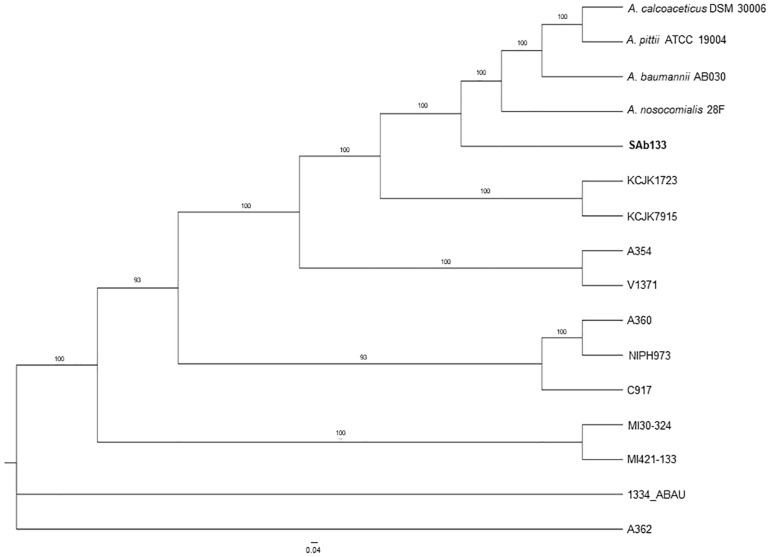
Phylogenetic tree of *A. seifertii* strains. A maximum likelihood phylogenetic tree representing 12 *A. seifertii* genomes (i.e., SAb133, KCJK7915, KCJK1723, 1334_ABAU, MI421-133, MI30-324, V1371, A354, A360, A362, C917, and NIPH973), *A. baumannii* AB030, *A. calcoaceticus* DSM 30006, *A. nosocomialis* 28F, and *A. pittii* ATCC 19004 based on concatenated alignment on 2,739.645 bp. *A. seifertii* SAb133 was highlighted in bold.

The comparison among the strains pangenomes revealed 2,152 orthologous clusters shared by at least two lineages. These clusters were composed by 59,215 homologous genes (totalizing 1,973,525 bp in length), which were joined end-to-end and concatenated in a unique FASTA file resulting in 73,593 distinct patterns as well as 2,830,60 parsimony-informative sites, 1,749,730 singleton sites and 1,515,492 constant sites used for phylogenetic reconstruction. The ML phylogeny revealed a well-structured dichotomy sand a clear separation in among all the strains (supported by bootstrap values higher than 93%), except for *A. seifertii* 1334-ABAU and *A. seifertii* A362 strains, which formed a polytomy. In this analysis, *A. baumannii* AB030, *A. pittii* ATCC 19004, *A. calcoaceticus* DSM 30006 and *A. nosocomialis* 28F were used as out-groups to improve the power of comparison and their phylogenetic position on the tree showed that the *A. seifertii* SAb133 strain is closely related to them even it forming a separated clade (Figure 1).

It was also observed that the *A. seifertii* SAb133 strain appears to be a transition between the *A. seifertii* clade and *A. calcoaceticus-A. baumannii* clade strains since it forms an intermediate group among the strains belonging to these taxa, being evolutively near to *A. baumannii* AB030 and the *A. seifertii* KCJK1723 and *A. seifertii* KCJK7915 taxa (Figure 1). At the same time, since the tree ramifications were sorted by increase of evolutive distances (mutation rates), the *A. seifertii* SAb133 strain presented as the lowest derivate strain, being the *A. seifertii* MI30-324 and *A. seifertii* MI421-133 the most derivate strains inside the *A seifertii* species. Unfortunately, for the *A. seifertii* 1334-ABAU and *A. seifertii* A360 strains there were not enough phylogenetic signals to uncover their phylogenetic position on the tree, justifying the polytomic position of these two *A. seifertii* strains on the tree (Figure 1).

**FIGURE 2:**
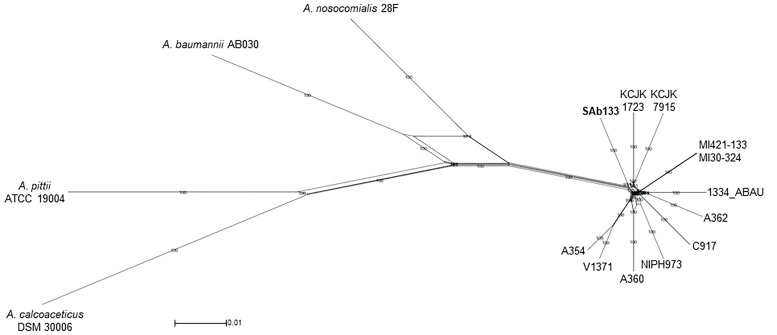
Neighbor-Net phylogenetic network of *A. seifertii* strains. A Neighbor-Net phylogenetic network representing 12 *A. seifertii* genomes (i.e., SAb133, KCJK7915, KCJK1723, 1334_ABAU, MI421-133, MI30-324, V1371, A354, A360, A62, C917, and NIPH973), *A. baumannii* AB030, *A. calcoaceticus* DSM 30006, *A. nosocomialis* 28F and *A. pittii* ATCC 19004 based in the concatenated alignment on 2,739.645 bp. *A. seifertii* SAb133 was highlighted in bold.

The same concatenated joined end-to-end 1,973,525 bp sequences were used to build a distance-based network using Neighbor-net algorithm. The topology of the network was an equal angle tree that supported the ML tree revealing a close relationship among *A. seifertii* SAb133 and the other strains (i.e., *A. seifertii* KCJK1723 and *A. seifertii* KCJK7915) as already inferred by phylogenetic analysis. Similarly, *A. baumannii* AB030, *A. pittii* ATCC 19004, *A. calcoaceticus* DSM 30006 and *A. nosocomialis* 28F strains were over again arranged as external groups (Figure 2). In addition, the proximity of clades of the *A. seifertii* SAb133 and *A. seifertii* A354/V1371, and *A. seifertii* MI30-324/MI421-133 to the *A. seifertii* 1334-ABAU was not coherent to the ML phylogenetic tree obtained (Figures 1, 2). This may indicate that possible conflicting signals shall be present among these strains (i.e., as differential evolutive rates or independent horizontal gene transfer events), which confound the phylogenetic history reconstruction.

### Genome Similarity

**FIGURE 3:**
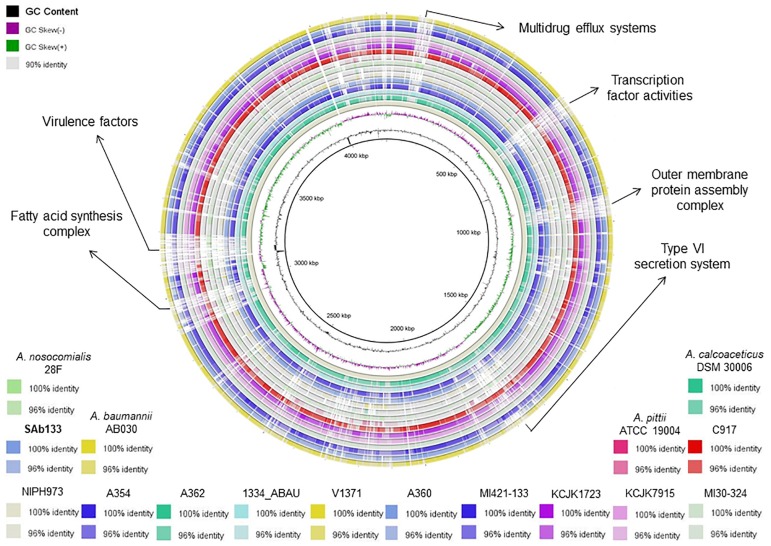
Blastmap comparison of *A. seifertii* strains. A circular comparison of 12 *A. seifertii* genomes (i.e., SAb133, KCJK7915, KCJK1723, 1334_ABAU, MI421-133, MI30-324, V1371, A354, A360, A362, C917, and NIPH973), *A. baumannii* AB030, *A. calcoaceticus* DSM 30006, *A. nosocomialis* 28F and *A. pittii* ATCC 19004. The genomes and sequence similarity (100, 96, and 90%) are demonstrated in different colors. *A. seifertii* NIPH973 was used as reference genome and *A. seifertii* SAb133 was highlighted in bold.

The ANI values among coding regions of the 12 *A. seifertii* strains were higher than 95% with little variations related to the strain-specific features that confer unique traits to a given strain. The comparison with *A. baumannii* AB030, *A. pittii* ATCC 19004, *A. calcoaceticus* DSM 30006 and *A. nosocomialis* 28F strains enhanced the differentiation of *A. seifertii* strains relatedness from bacteria belonging to *A. calcoaceticus-A. baumannii* complex (Table 2). A Blasmap was constructed for visualizing the synonymy in ANI values between those strains and the map showed the high degree of synonymy between *A. seifertii* strains and the non-synonymy among the *A. seifertii* strains and *A. baumannii* AB030, *A. pittii* ATCC 19004, *A. calcoaceticus* DSM 30006 and *A. nosocomialis* 28F strains. Some hypervariable genomic regions were also detected, such as multidrug efflux pumps, transcription factor activities, outer membrane protein assembly complex, type VI secretion system, fatty acid synthesis complex and virulence factors (Figure 3).

**TABLE 2:** ANI values among *Acinetobacter* sp.

	**Strains**	**(1)**	**(2)**	**(3)**	**(4)**	**(5)**	**(6)**	**(7)**	**(8)**	**(9)**	**(10)**	**(11)**	**(12)**	**(13)**	**(14)**	**(15)**	**(16)**
(1)	*A. baumannii* AB030	^∗^															
(2)	*A. calcoaceticus* DSM 30006	87.15	^∗^														
(3)	*A. nosocomialis* 28F	91.96	87.22	^∗^													
(4)	*A. pittii* ATCC 19004	88.84	90.17	88.45	^∗^												
(5)	1334_ABAU	90.27	87.56	92.19	88.77	^∗^											
(6)	A354	90.29	87.37	92.13	88.63	**97.05**	^∗^										
(7)	A360	90.26	87.47	92.08	88.74	**96.75**	**97.21**	^∗^									
(8)	A362	90.25	87.37	92.43	88.68	**97.11**	**96.94**	**96.73**	^∗^								
(9)	C917	90.38	87.53	92.23	88.73	**96.79**	**96.84**	**96.68**	**96.94**	^∗^							
(10)	KCJK1723	90.21	87.57	92.04	88.78	**97.01**	**96.79**	**96.66**	**96.96**	**96.75**	^∗^						
(11)	KCJK7915	90.28	87.51	92.11	88.71	**96.93**	**96.74**	**96.73**	**96.99**	**96.66**	**97.19**	^∗^					
(12)	MI30-324	90.39	87.51	92.16	88.86	**97.09**	**96.97**	**96.82**	**97.13**	**96.91**	**97.06**	**96.93**	^∗^				
(13)	MI421-133	90.45	87.51	92.20	88.86	**97.07**	**96.92**	**96.84**	**97.12**	**96.91**	**97.05**	**96.95**	**99.95**	^∗^			
(14)	NIPH973	90.25	87.42	92.01	88.69	**96.93**	**96.89**	**96.94**	**96.78**	**96.76**	**96.93**	**96.78**	**96.96**	**96.97**	^∗^		
(15)	SAb133	90.34	87.57	92.25	88.85	**96.78**	**97.01**	**96.83**	**96.82**	**96.83**	**96.90**	**96.79**	**96.77**	**96.77**	**96.73**	^∗^	
(16)	V1371	90.32	87.45	92.17	88.69	**96.94**	**98.49**	**97.26**	**96.86**	**96.87**	**96.81**	**96.85**	**96.96**	**96.97**	**96.81**	**97.02**	^∗^

*The values in bold show >95% threshold for species assignment. The asterisk (^∗^) indicates 100% of similarity as the genomes.*

### Constitution of Pangenomes

The pangenome analysis among the 12 *A. seifertii* strains (Table 1) returned 6,522 orthologous clusters that are representing the *A. seifertii* pangenome’s constitution. In the light of these definitions, from the 6,522 orthologous clusters, a total of 2,860 soft-core genes, 2,458 core genes, 2,722 cloud genes, and 940 shell genes were characterized. Novel genomic regions related to biological processes category were detected in *A. seifertii* strains cloud genomes, such as drug transmembrane transport (i.e., C917, A362, and KCJK1723 – 25% of prevalence), siderophore transport (i.e., 1334-ABAU, A354, and A362 – 25% of prevalence), efflux transmembrane transporter activity (i.e., C917 and KCJK 1723 – 17% of prevalence), and β-lactamase activity and β-lactam antibiotic catabolic process (SAb133 strain only – 8,3% of prevalence). In addition, since the biological process “DNA restriction-modification systems” was found in at least 50% of the strains (i.e., SAb133, C917, 1334-ABAU, A354, A360, A362, and V1371), this process may be more related to the shell genome composition than a cloud genome metabolic process.

### Genomic Islands and Phage-Related Sequences

*Acinetobacter seifertii* SAb133 presented 15 genomic islands ranged in size from 4237 bp to 32680 bp (average 7928 ± 7153 bp). The largest genomic island (32680 bp) identified in *A. seifertii* SAb133 was related to virulence and antimicrobial resistance mechanisms (Supplementary Table S1). No Phage-related sequence was detected in *A. seifertii* SAb133 and the other *A. seifertii* strains had at least one region containing Phage-related sequence, except the KCJK7915 (Supplementary Table S2). Eight genomic islands containing Phage-related sequences were interspersed throughout the genomes of *A. seifertii* strains (Supplementary Table S1).

### Resistome, Virolome and Mobile Elements

Resistome analysis showed antimicrobial resistance genes for β-lactams (*bla*_ADC–__25_ and *bla*_TEM_) and multidrug efflux systems (RND, MFS, MATE, and SMR). Among RND and MFS systems, *A. seifertii* SAb133 presented the families AdeB/AdeJ and MdtB/MuxB, and Bcr/CflA and DHA2, respectively. Mutation in 23S rRNA (A2058G) that confers resistance to erythromycin, azithromycin and telithromycin were also detected. A great diversity of metal tolerance genes were detected, including for copper (*copC, copD, corC*, and *nlpE*), arsenic (*arsB, arsC*, and *arsH*), magnesium/cobalt (*corA* and *mgtA*), cadmium/zinc/cobalt (*cusA* and *czcA*), nickel/cobalt (*nreB*) and tellurite/selenite (*ruvB*), chromium (*chrA*), and tellurium (*terD*). The SAb133 isolate carried ABKVGs, including *barA* (acinetobactin), *pmrB* (sensor kinase), *omp*A (adherence), *pbpG* (serum resistance), *pgaABCD* and *csu pili* (biofilm formation), and *galU* (immune evasion) (Table 3).

**TABLE 3:** Detection of ARGs, CPMs, multidrug efflux systems, MTGs, ABKVGs, ISs, and plasmids among *A. seifertii* strains.

**Strains**	**ARGs^a^**	**CPMs^b^**	**Multidrug**	**MTGs^c^**	**ABKVGs^d^**	**ISs^e^**	**Plasmids**
		**of 23S rRNA**	**efflux systems**				
SAb133	*bla*_ADC–__25_, *bla*_TEM_	A2058G	RND, MFS, MATE, SMR	*copC, copD, corC, nlpE, arsB, arsC, arsH, corA, mgtA, cusA, czcA, nreB, ruvB, chrA, terD*	*barA, ompA, pbpG, pgaABCD, csu pili, pmrB, galU*	IS3, IS5, Tn3	GR2, GR6
KCJK7915	*bla*_ADC–__25_	A2058G	RND, MFS, MATE, SMR	*copB, corC, nlpE, arsB, arsC, arsH, corA, mgtA, cusA, czcA, ruvB, terD*	*barA, pgaABCD, ompA, galU, csu pili, ptk*	IS3	GR2, GR6
KCJK1723	*bla*_ADC–__25_	A2058G	RND, MFS, MATE, SMR	*copB, nlpE, ruvB*	*barA, pgaABD, csu pili, ptk*	IS3	GR2, GR6
1334_ABAU	*bla*_ADC–__25_	A2058G	RND, MFS, MATE, SMR	*copB, copC, copD, corC, nlpE*, *arsC, corA, cusA, czcA, nreB, ruvB*	*pgaABD, ompA, galU*	IS5	GR2, GR6
MI421-133	*bla*_ADC–__25_	A2058G	RND, MFS, MATE, SMR	*copB, corC, nlpE, arsB, arsC, arsH, corA, mgtA, cusA, czcA, ruvB*	*barA, pgaABCD, ompA, galU, csu pili, ptk*	ISA110	GR2, GR6
MI30-324	*bla*_ADC–__25_	A2058G	RND, MFS, MATE, SMR	*copB, corC, nlpE, arsB, arsC, arsH, corA, mgtA, cusA, czcA, ruvB*	*barA, pgaABCD, ompA, galU, csu pili, ptk*	ISA110	GR2, GR6
V1371	*bla*_ADC–__25_	A2058G	RND, MFS, MATE, SMR	*copB, corC, nlpE, arsB, arsC, arsH, corA, mgtA, cusA, czcA, ruvB*	*barA, pgaABCD, ompA, galU, ptk*	IS21	GR2, GR6
C917	*bla*_ADC–__25_	A2058G	RND, MFS	*copC, corC, nlpE, arsH, corA, mgtA, czcA, ruvB, chrA*	*ompA, csu pili*	IS3, IS5, IS6, IS66, IS256, ISNCY	GR2, GR6, GR7
A354^f^	*bla*_ADC–__25_	A2058G	RND, MFS	*copB, nlpE, arsB, arsH, ruvB*	*pgaABCD, ompA, csu pili, lpsB, pmrB, pbpG, eps, ptk*	IS3, IS5, IS66, Tn3	GR2, GR6, GR7, ColRNAI
A360^f^	*bla*_ADC–__25_	A2058G	RND, MFS	*copB, copC, copD, nlpE, arsB, arsC, arsH, czcA, ruvB*	*pgaABCD, ompA, csu pili, lpsB, pmrB, pbpG, eps, ptk*	IS3, IS5, IS66, IS256, ISNCY	GR2, GR6, GR7
A362^f^	*bla*_PER–__1_, *aacA4, sul1, sul2, aph(3′)-VIa, aac(6′)Ib-cr, msr(E), mph(E), aac(3)-IId, floR, ARR-3*	A2058G	RND, MFS	*copB, copC, copD, nlpE, arsB, arsH, ruvB*	*pgaABCD, ompA, csu pili, lpsB, pmrB, pbpG, eps, ptk*	IS1, IS3, IS5, IS30, IS66, IS91, IS256, ISNCY	GR2, GR6, GR7, ColRNAI
NIPH973	*bla*_ADC–__25_	A2058G	RND, MFS	*nlp*E, *arsH, corA, ruvB*	*pgaABD*	IS1, IS3, IS4, IS5, IS6, IS30, IS66, IS256, IS630, ISNCY	GR2, GR6, GR7

*^*a*^ARGs, antimicrobial resistance genes. ^*b*^CPM, chromosomal point mutations; A, adenine; G, guanine. ^*c*^MTGs, metal tolerance genes. ^*d*^ABKVGs, *A. baumannii* known virulence genes. ^*e*^ISs, insertion elements. ^*f*^Data from Yang et al. (2016) expect for chromosomal point mutations, multidrug efflux pump systems, metal tolerance genes, insertion elements, and plasmids.*

For comparing, ARGs, CPMs, MTGs, and ABKVGs detected in the *A. seifertii* SAb133 were analyzed in the 11 *A. seifertii* strains (i.e., KCJK7915, KCJK1723, 1334_ABAU, MI421-133, MI30-324, V1371, C917, A354, A360, A362, and NIPH973) using the genome annotation available in GenBank (Table 1). All *A. seifertii* strains presented *bla*_ADC–__25_, mutations in 23S rRNA (A2058G) and multidrug efflux systems (RND and MFS). Among RND efflux systems, the majority of *A. seifertii* strains (i.e., 1334_ABAU, KCJK7915, KCJK1723, MI421-133, MI30-324, and V1371) presented the AdeA/*Ade*I family and four *A. seifertii* strains (i.e., SAb133, 1334_ABAU, MI421-133, and MI30-324) presented the AdeB/AdeJ family. Several MTGs and ABKVGs have also detected in *A. seifertii* strains, principally in strains obtained from the environment and human (i.e., KCJK7915, MI421-133, MI30-324, V1371, A354, A360, A362, and C917). All *A. seifertii* strains presented at least ARG, CPM, MTG, and ABKVG (Table 3).

*Acinetobacter seifertii* SAb133 was the only one that presented *bla*_TEM_, RND efflux system (MdtB/MuxB family) and MFS efflux system (Bcr/CflA and DHA2 families) (Table 3). Two MTGs (*nreB* and *terD*) were detected only in three *A. seifertii* strains (i.e., SAb133, KCJK7915, and 1334_ABAU). The SAb133 genome presented three IS elements (i.e., IS3, IS5, and Tn3) and the all other *A. seifertii* strains had a least one IS element (Table 3). All *A. seifertii* strains presented GR2 and GR6 plasmids, and five *A. seifertii* strains (i.e., C917, A354, A360, A362, and NIPH973) presented the GR7. Curiously, the ColRNAI, a plasmid commonly detected in *Enterobacteriaceae*, was detected in A354 and A362 strains (Table 3). In addition, *A. seifertii* SAb133 also had the highest amount of genes related to antimicrobial resistance, tolerance to metals, and *A. baumannii* known virulence factors, except for Chinese clinical isolates (i.e., A354, A360, and A362).

### Epidemiological Analysis

**FIGURE 4:**
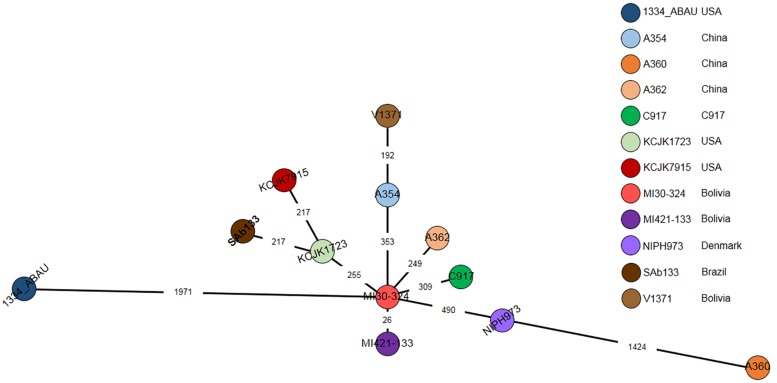
cgMLST of 12 *A. seifertii* strains based on BacWGSTdb. Each circle represents an *A. seifertii* strain. The numbers on the connecting lines show the number of locus mismatches between *A. seifertii* strains. *A. seifertii* SAb133 was highlighted in bold.

A cgMLST based comparison identified from 23 to 1971-loci variant among *A. seifertii* strains. The smallest difference (26-loci variant) occurred between *A. seifertii* MI30-324 and *A. seifertii* MI421-133, both from Bolivia, while the largest difference (1971-loci variant) occurred between *A. seifertii* MI30-324 (Bolivia) and *A. seifertii* 1334_ABAU (United States). Five other *A. seifertii* strains (i.e., KCJK1723, A354, A362, C917, and NIPH973) belonging to China, United States and Dernmark also differed by *A. seifertii* MI30-324 from 255 to 490-loci variant. *A. seifertii* SAb133 (Brazil) and *A. seifertii* KCJK7915 (United States) differed by *A. seifertii* KCJK1723 (United States) by 213-loci variant and *A. seifertii* V1371 (Bolivia) differed by *A. seifertii* A354 (China) by 192-loci variant (Figure 4).

## Discussion

*Acinetobacter calcoaceticus-Acinetobacter baumannii* complex has phenotypic and genotypic similar species and the WGS has proved to be an useful tool for identification of closely related species. To support species assignments, >95% threshold is commonly applied, which was detected in this study, supporting that SAb133 isolate belongs to *A. seifertii* (Richter and Rosselló-Móra, 2009; Varghese et al., 2015). Phylogenetic analysis showed that all *A. seifertii* strains were equally distributed when compared with non-*A. seifertii* strains. Thus, it is possible to suggest a phylogenetic history characterized by several independent evolutive processes (e.g., differential evolution rates among the strains and differential selection by several stress factors), due to the differences in the topologies of the phylogenetic Neighbor-Net tree and the ML tree, high lightening possible conflicts of phylogenetic signals which make harder the evolutive history elucidation of some *A. seifertii* strains.

The bacterial pangenome is defined as the sum of the core and accessory genomes (i.e., shell and cloud) and the core genome comprises the essential gene families sequenced in all bacterial species of a given clade. In addition, a fraction of the core genome may be split in soft-core, which refer to the set of genes present in at least 95% of all strains. The accessory genomes represent the set of non-essential genes present in a restrict number of strains. The shell genome represents a dispensable set of genes relatively ubiquitous in some taxa, while the cloud genome is related to a restrict set of genes present in very few strains (Contreras-Moreira and Vinuesa, 2013; McInerney et al., 2017). The pangenome results showed differential biological processes associated with survival and adaptation of *A. seifertii* strains, which can be related to their diverse physiological capabilities due to their ubiquitous distribution (i.e., human, animal, and environment) (Cordero and Polz, 2014).

Antimicrobial resistance is a worldwide public health threat and MDR bacteria have been reported in soil samples, despite the scarce number of papers addressing this topic in this environment. OXA-48-producing *A. seifertii* strains were recently reported in Brazil, with OXA-type β-lactamases being the most frequent ARG described in *Acinetobacter* sp., mainly in *A. baumannii* (Cayô et al., 2016; Narciso et al., 2017). Extended-spectrum AmpC cephalosporinase (ADC-25), and *A. baumannii* known virulence genes have been described in *A. seifertii* clinical strains (Yang et al., 2016), and TEM β-lactamase and intrinsic acquired efflux pumps (RND and non-RND) have also been reported in *Acinetobacter* spp. conferring resistance to different antimicrobials, antiseptics, biocides and detergents (Coyne et al., 2011; Montaña et al., 2016). *A. seifertii* SAb133 presented different antimicrobial resistance markers, which are closely related to the MDR phenotype found.

Metals are widely used as a growth promoter in animals and, consequently, may be biomagnified in the environment. The presence of metal compounds in the environment can select bacteria with reduced susceptibility to these compounds, leading to co-selection and reduced antimicrobial susceptibility (Wales and Davies, 2015). *A. seifertii* SAb133 showed several MTGs, which are associated with high MICs for metal compounds. The presence of antimicrobial resistance and metal tolerance genes in several bacterial genera including *Acinetobacter* sp., have been increasingly reported, which is of concern (Ji et al., 2012; Seiler and Berendonk, 2012).

The WGS has been used in different types of bacterial analysis, including evolution, epidemiology, pathogenicity and antimicrobial resistance (Almeida and De Martinis, 2019), and WGS-based typing of bacteria has been increasingly used for investigation of outbreaks and surveillance studies (Ruan and Feng, 2016). Different relatedness criteria for cgMLST were described for representative clinically relevant bacteria, including NFGNB, *Enterococcus* sp. and *Mycobacterium* sp. and enterobacteria. To date, among the species belonging to the *A. calcoaceticus-A. baumannii* complex, the *A. baumannii* is the only one to have an established relatedness criterion for cgMLST (≤3-loci) (Higgins et al., 2017; Schürch et al., 2018). Therefore, based on relatedness criteria for cgMLST of *A. baumannii*, the great majority of *A. seifertii* strains presented large amounts of locus differences, showing the divergence between these species.

Genomic islands are clusters of genes involved in the genome evolution and microbial adaptability, which can be classified into different subtypes, such as metabolic, fitness, symbiotic, antimicrobial resistance, and pathogenicity (Juhas et al., 2009). The mobile genetic elements (e.g., pathogenicity islands, plasmids, transposons, and insertion sequences) are closely related to the rapid development of MDR *Acinetobacter* sp., mainly *A. baumannii*. A great diversity of plasmids has been described in *A. baumannii*, which represent powerful routes for the evolution of antimicrobial resistance (Peleg et al., 2008; Lean and Yeo, 2017).

## Conclusion

This study reports the first whole genome sequence of an *A. seifertii* isolated from a soil sample. To the best of our knowledge, this is the third report in Brazil and the fourth in the Latin America of an *A. seifertii*, being all the previous reports on isolates recovered from human and animal infections. The comparative genome sequence analysis done in this study showed that SAb133 presented the highest amount of determinants related to antimicrobial resistance and tolerance to metals, beyond the presence of *A. baumannii* known virulence genes, which demonstrates the great virulence potential derived from environmental *A. seifertii* strains. Moreover, this study contributes to a better understanding of the genetic relationship among the few known *A. seifertii* strains worldwide distributed.

## Data Availability

The datasets generated for this study can be found in the GenBank accession no. SNSA00000000.

## Author Contributions

JF isolated the bacterium and performed the phenotypic analyses. OA performed the phylogenetic analyses. JF and OA conceptualized the study, performed the comparative genomic analyses, and drafted the manuscript. ES and ED coordinated the project and revised the manuscript. All the authors approved the final manuscript.

## Conflict of Interest Statement

The authors declare that the research was conducted in the absence of any commercial or financial relationships that could be construed as a potential conflict of interest.
