# Supplementary material for: Characterization of an Environmental Multidrug-Resistant Acinetobacter seifertii and Comparative Genomic Analysis Reveals Co-occurrence of Antimicrobial Resistance and Metal Tolerance Determinants
Source: Front Microbiol. 2019 Sep 18;10:2151. doi: 10.3389/fmicb.2019.02151 (PMC6759475; doi:10.3389/fmicb.2019.02151)
Supplement: Supplementary file 1 [file Table_1.DOCX]

**Supplementary Table 1 -** Genomic islands detected in *A. seifertii* strains.

| **Strain** | **Genomic Island (GI)** | **Region Length (bp)** | **Product** |
| --- | --- | --- | --- |
| **SAb133** | GI-1 | 5608 | Type VI secretion system tip protein VgrG, LysM peptidoglycan-binding domain-containing protein, SH3 domain-containing protein, DUF1311 domain-containing protein, hypothetical proteins |
|  | GI-2 | 9912 | TetR/AcrR family transcriptional regulator, aldo/keto reductase, AraC family transcriptional regulator, carboxymuconolactone decarboxylase family protein, transcriptional regulator, flavodoxin family protein, DUG3696 domain-containing protein, hypothetical proteins |
|  | GI-3 | 6087 | Type I secretion C-terminal target domain-containing protein, hypothetical proteins |
|  | GI-4 | 4523 | DUF2750 domain-containing proetin, HNH endonuclease, IS3 family transposase, helix-turn-helix domain-containing protein, hypothetical proteins |
|  | GI-5 | 11185 | TetR/AcrR family transcriptional regulator, acetyltransferase, HD domain-containing protein, hydrolase, DMT family transporter, bifunctional DNA-binding transcriptional regulator/O6-methylguanine-DNA methyltransferase Ada, SDR family oxidoreductase, hypothetical proteins |
|  | GI-6 | 5120 | TetR/AcrR family transcriptional regulator, IS3 family transposase, helix-turn-helix domain-containing protein, hypothetical proteins |
|  | GI-7 | 6626 | TetR/AcrR family transcriptional regulator, HD domain-containing protein, DNA-binding protein, PAAR domain-containing protein, hypothetical proteins |
|  | GI-8 | 5321 | Phenylacetic acid degradation protein, histidine phosphatase family protein, hypothetical proteins |
|  | GI-9 | 4305 | TetR/AcrR family transcriptional regulator, sel1 repeat family protein, SRPBCC family protein, hypothetical proteins |
|  | GI-10 | 4237 | TetR/AcrR family transcriptional regulator, IS3 family transposase, alpha/beta hydrolase, MarR family transcriptional regulator, helix-turn-helix domain-containing protein |
|  | GI-11 | 8193 | AlpA family transcriptional regulator, DUF1569 domain-containing protein, molecular chaperone DnaJ, hypothetical proteins |
|  | GI-12 | 6532 | MFS transporter, TIGR02391 family protein, outer membrane porin OprD family, hypothetical proteins |
|  | GI-13 | 4041 | Site-specific integrase, hypothetical protein |
|  | GI-14 | 4992 | LysR family transcriptional regulator, LysE family translocator, AraC family transcriptional regulator, EamA family transporter, hypothetical protein |
|  | GI-15 | 32680 | Type II secretion protein F, PEP-CTERM sorting domain-containing protein, CpaF family protein, glucan biosynthesis glucosyltransferase H, carboxylesterase, ribonucleoside-diphosphate reductase subunit alpha, GNAT family N-acetyltransferase, NAD-dependent DNA ligase LigA, flagellar protein, class A beta-lactamase, cytochrome o ubiquinol oxidase subunit I, MATE family efllux transporter, bifunctional nicotinamite-nucleotide adenylyltransferase/Nudix hydrolase, nicotinate phosphoribosyltransferase, hypothetical protein |
| **KCJK7915** | GI-1 | 7608 | SH3 domain-containing protein, esterase, hypothetical proteins |
|  | GI-2 | 5050 | TetR Family transcriptional regulator, peptidase M15, hypothetical proteins |
|  | GI-3 | 7847 | TetR Family transcriptional regulator, hypothetical proteins |
|  | GI-4 | 4163 | Stress-induced protein, hypothetical proteins |
|  | GI-5 | 5000 | SRPBCC family protein, TetR Family transcriptional regulator, serine hydrolase, hypothetical proteins |
|  | GI-6 | 6327 | VOC family protein, branched-chain amino acid ABC transporter permeasse, peptidase, hypothetical proteins |
|  | GI-7 | 5257 | Darcynin 1, TetR Family transcriptional regulator, hypothetical proteins |
|  | GI-8 | 10484 | Molecular chaperone DnaJ, IS66 family transposase, integrase, hypothetical proteins |
|  | GI-9 | 32587 | PEP-CTERM sorting domain-containing protein, hypothetical proteins |
|  | GI-10 | 7958 | RTX toxin, leukotoxin, hypothetical proteins |
|  | GI-11 | 11280 | Flagellar hook-length control protein FliK, hemolysin, ATP-binding protein, hypothetical proteins |
|  | GI-12 | 4468 | Flagellar protein, hypothetical proteins |
|  | GI-13 | 5544 | Flagellar hook-length control protein FliK, adhesion, SMI1/KNR4 family protein, hypothetical proteins |
|  | GI-14 | 16110 | Flagellar hook-length control protein FliK, flagellar protein, oxidoreductase, cytoplasmatic protein, DUF1311 domain-containing protein, hypothetical proteins |
|  | GI-15 | 12232 | Flagellar hook-length control protein FliK, hypothetical proteins |
|  | GI-16 | 6118 | Flagellar hook-length control protein FliK, biofilm associated protein, hypothetical proteins |
|  | GI-17 | 12151 | Flagellar hook-length control protein FliK, biofilm associated protein, Efflux transporter periplasmatic adaptor subunit, YqaJ-like viral recombinase, hypothetical proteins |
|  | GI-18 | 7368 | Hemolysin, adhesin, hypothetical proteins |
|  | GI-19 | 5162 | RTX toxin, adhesin, hypothetical proteins |
| **KCJK1723** | GI-1 | 10355 | TetR family transcriptional regulator, acetyltransferase, hydrolase, hypothetical proteins |
|  | GI-2 | 4423 | DNA polymerase V, DNA polymerase V subunit UmuC, ACR protein, hypothetical proteins |
|  | GI-3 | 5704 | Hypothetical proteins |
|  | GI-4 | 8613 | Beta-ketoacyl-[acyl-carrier-protein] synthase I, GNAT family acetyltransferase, hypothetical proteins |
|  | GI-5 | 8700 | Type II secretion protein E, hemolysin, biofilm associated protein, hypothetical proteins |
|  | GI-6 | 7416 | MFS transporter, hypothetical proteins |
|  | GI-7 | 4712 | Biofilm associated protein, transcriptional regulator, hypothetical proteins |
|  | GI-8 | 19802 | Biofilm associated protein, glycolate oxidase subunit GlcD, hypothetical proteins |
|  | GI-9 | 8338 | Haloacid dehalogenase, hypothetical proteins |
| **1334_ABAU** | GI-1 | 8789 | Bifunctional transcriptional activator/DNA repair enzyme Ada, hypothetical proteins |
|  | GI-2 | 5188 | IS3 family transposase ISAba22, hypothetical proteins |
|  | GI-3 | 4638 | Transcriptional repressor PaaX, Protein YrdA, FMN-dependent NADH-azoreductase, hypothetical proteins |
|  | GI-4 | 9723 | Alcohol dehydrogenase, hypothetical proteins |
|  | GI-5 | 6709 | Replicative DNA helicase, hypothetical proteins |
|  | GI-6 | 9220 | Dimodular nonribosomal peptide synthase, Polyketide synthase PksN, 2-succinylbenzoate--CoA ligase, Transcriptional activator protein AnoR, Acyl-homoserine-lactone synthase, hypothetical proteins |
|  | GI-7 | 10955 | 3-oxoacyl-[acyl-carrier-protein] synthase 1, hypothetical proteins |
|  | GI-8 | 8708 | UvrABC system protein B, hypothetical proteins |
|  | GI-9 | 8584 | Hypothetical proteins |
|  | GI-10 | 9457 | DNA primase, Protease HtpX, hypothetical proteins |
|  | GI-11 | 17320 | Glucose-1-phosphate thymidylyltransferase 1, dTDP-4-dehydrorhamnose 3,5-epimerase, dTDP-4-dehydrorhamnose reductase, Putative O-antigen transporter, UDP-N-acetylglucosamine 2-epimerase, hypothetical proteins |
|  | GI-12 | 11595 | ATP-dependent Clp protease ATP-binding subunit ClpC, Thioredoxin 2, hypothetical proteins |
|  | GI-13 | 5976 | Alkanesulfonate monooxygenase, hypothetical proteins |
|  | GI-14 | 7158 | Hypothetical proteins |
|  | GI-15 | 10509 | Hypothetical proteins |
| **MI421-133** | GI-1 | 5636 | TetR family transcriptional regulator, EamA Family transporter, bifunctional DNA-binding transcriptional regulator/O6-methylguanine-DNA methyltransferase Ada, short-chain dehydrogenase, hypothetical proteins |
|  | GI-2 | 9936 | Restriction endonuclease subunit R, SAM-dependent DNA methyltransferase, restriction endonuclease subunit S, hypothetical proteins |
|  | GI-3 | 6556 | TetR family transcriptional regulator, transcription elongation factor GreB, tautomerase, hypothetical proteins |
|  | GI-4 | 4086 | Type IV secretion protein Rhs, DUF4882 domain-containing protein, glyoxalase, hypothetical proteins |
|  | GI-5 | 9151 | SMI1/KNR4 family protein, cupin, hypothetical proteins |
|  | GI-6 | 6556 | SMI1/KNR4 family protein, fimbrial biogenesis outer membrane usher protein, hypothetical proteins |
| **MI30-324** | GI-1 | 4174 | AraC family transcriptional regulator, aldo/keto reductase, hypothetical proteins |
|  | GI-2 | 5677 | Hypothetical proteins |
|  | GI-3 | 4164 | Hypothetical proteins |
|  | GI-4 | 4860 | SMI1/KNR4 family protein, hypothetical proteins |
|  | GI-5 | 9936 | SAM-dependent DNA methyltransferase, restriction endonuclease subunit S, hypothetical proteins |
|  | GI-6 | 4943 | TetR/AcrR family transcriptional regulator, darcynin 1, cell division protein ZapE, chromosome replication initiation inhibitor protein, hypothetical proteins |
|  | GI-7 | 7695 | Hypothetical proteins |
|  | GI-8 | 18476 | SMI1/KNR4 family protein, outer membrane assembly protein BamE, hemagglutinin, hypothetical proteins |
|  | GI-9 | 8554 | SMI1/KNR4 family protein, transcription elongation factor GreB, tautomerase, rhodanese, hypothetical proteins |
|  | GI-10 | 8003 | LysR family transcriptional regulator, glutathione transferase GstA, transcription elongation factor GreB, MFS transporter, hypothetical proteins |
| **V1371** | GI-1 | 5062 | DUF1311 domain-containing protein, esterase, hypothetical proteins |
|  | GI-2 | 14506 | TetR/AcrR family transcriptional regulator, AraC family transcriptional regulator, aldo/keto reductase, CAAX protease, TetR/AcrR family transcriptional regulator, NAD(P)H oxidoreductase, NAD(P)H oxidoreductase, EamA family transporter, bifunctional DNA-binding transcriptional regulator/O6-methylguanine-DNA methyltransferase Ada, short-chain dehydrogenase, hypothetical proteins |
|  | GI-3 | 4480 | Glutamate synthase large subunit |
|  | GI-4 | 4404 | Hypothetical proteins |
|  | GI-5 | 4642 | YqaJ-like viral recombinase, hydrolase or metal-binding protein, AlpA family phage regulatory protein, class I SAM-dependent methyltransferase, HNH endonuclease, hypothetical proteins |
|  | GI-6 | 6252 | TetR family transcriptional regulator, sel1 repeat family protein, SRPBCC family protein, urea carboxylase, hypothetical proteins |
|  | GI-7 | 4214 | Helicase, hypothetical proteins |
|  | GI-8 | 4101 | Plasmid replicase, hypothetical proteins |
|  | GI-9 | 8042 | Chromosome partitioning protein ParB, ParA family protein, flagellar protein, hypothetical proteins |
|  | GI-10 | 14084 | RNA-NAD 2'-phosphotransferase, RepB family plasmid replication initiator protein, hypothetical proteins |
|  | GI-11 | 4015 | DNA-binding protein, phosphohydrolase, hypothetical protein, hypothetical proteins |
|  | GI-12 | 16672 | XRE family transcriptional regulator, N-6 DNA methylase, chromosome partitioning protein ParB, hypothetical proteins |
| **C917** | GI-1 | 12109 | SH3 domain protein, LysM domain protein, hypothetical proteins |
|  | GI-2 | 6887 | Phage integrase, integrase, Transcriptional activator protein phzR, hypothetical protein |
|  | GI-3 | 6024 | Phenylacetic acid degradation operon negative regulatory protein, PaaY, phenylacetic acid degradation protein, alpha-ribazole-5'-phosphate phosphatase, major facilitator superfamily MFS_1, Phytanoyl-CoA dioxygenase, uroporphyrin-III C/tetrapyrrole methyltransferase, hypothetical proteins |
|  | GI-4 | 8836 | Putative HTH-like transcriptional regulator, phage-related protein, dichlorophenol hydroxylase, phage-like protein, hypothetical proteins |
|  | GI-5 | 12467 | Transposase, hypothetical proteins |
|  | GI-6 | 7004 | Methionine-R-sulfoxide reductase, methionine sulfoxide reductase A, diguanylate cyclase with GAF sensor, peptide methionine sulfoxide reductase, alkylhydroperoxidase, hypothetical proteins |
|  | GI-7 | 5051 | GNAT family acetyltransferas, hypothetical proteins |
|  | GI-8 | 10250 | Putative transcriptional regulator, phage/plasmid-related protein, hypothetical proteins |
|  | GI-9 | 4335 | Hypothetical proteins |
|  | GI-10 | 4029 | MerR family transcriptional regulator, deoxyribodipyrimidine photolyase-like protein, hypothetical proteins |
|  | GI-11 | 15937 | SMC domain-containing protein, integrase, putative alcohol dehydrogenase, zinc-containing, hypothetical proteins |
|  | GI-12 | 17585 | Transposase, CRO family transcriptional regulator, putative integrase, ATPase AAA-2 domain-containing protein, putative signal peptide protein, hypothetical proteins |
| **A354** | GI-1 | 9673 | Haloacid dehalogenase, hypothetical proteins |
|  | GI-2 | 4057 | TetR family transcriptional regulator, aldo/keto reductase, hypothetical proteins |
|  | GI-3 | 4242 | Phage head-tail adapter protein, hypothetical proteins |
|  | GI-4 | 14722 | TetR family transcriptional regulator, helicase, hypothetical proteins |
|  | GI-5 | 4674 | TetR family transcriptional regulator, XRE family transcriptional regulator, toxin HipA, hypothetical proteins |
|  | GI-6 | 5305 | TetR family transcriptional regulator, Sel1 repeat protein, polyketide cyclase, urea carboxylase, hypothetical proteins |
|  | GI-7 | 6309 | Transposase, antitoxin of toxin-antitoxin stability system N-terminal domain protein, ATPase AAA, DNA-binding protein, hypothetical proteins |
|  | GI-8 | 17177 | diguanylate cyclase (GGDEF) domain-containing protein, diguanylate cyclase, Zn-dependent protease, 2-alkenal reductase, diguanylate cyclase, sodium:proton exchanger, thioredoxin, Clp protease ClpC, heat-shock protein Hsp20, restriction endonuclease subunit M , hypothetical proteins |
|  | GI-9 | 4285 | 3-oxoacyl-ACP synthase, hypothetical proteins |
|  | GI-10 | 8736 | Cro/Cl family transcriptional regulator, toxin RelE, hypothetical proteins |
|  | GI-11 | 25944 | Micrococcal nuclease, RNA polymerase, nikA protein, transposase, hypothetical proteins |
|  | GI-12 | 8457 | Transposase, hypothetical proteins |
|  | GI-13 | 47795 | DNA primase, DNA helicase, beta-lactamase, spore coat protein CotH, DNA replication protein, lysozyme, terminasse, DNA-binding protein, glutaredoxin, ABC transporter, chromosome partitioning protein ParB, chromosome partitioning protein ParB, peptidoglycan-binding protein LysM, transposase, hypothetical proteins |
| **A360** | GI-1 | 9540 | Transposase, GNAT family acetyltransferase, carbamoxyltransferase, ABC transporter, hypothetical proteins |
|  | GI-2 | 4549 | Haloacid dehalogenase, hypothetical proteins |
|  | GI-3 | 7836 | Integrase, hypothetical proteins |
|  | GI-4 | 4129 | Phage/plasmid-like protein TIGR03299, YqaJ-like viral recombinase domain protin, hydrolase or metal-binding protein, hypothetical proteins |
|  | GI-5 | 4918 | TetR family transcriptional regulator, hypothetical proteins |
|  | GI-6 | 4260 | 2-alkenal reductase, transposase, hypothetical proteins |
|  | GI-7 | 4587 | Branched-chain amino acid permease, Branched-chain amino acid ABC transporter, transposon Tn21 resolvase, DNA-binding protein, phosphatidylinositol kinase, Cro/Cl family transcriptional regulator, hypothetical proteins |
|  | GI-8 | 17047 | ATPase AAA, antitoxin of toxin-antitoxin stability system N-terminal domain protein, DNA-binding protein, toxin-antitoxin system, toxin component, membrane protein, cell surface protein, transposase, hypothetical proteins |
|  | GI-9 | 9101 | TetR family transcriptional regulator, darcynin 1, Sel1 repeat protein, type IV secretion protein Rhs, hypothetical proteins |
|  | GI-10 | 8139 | Phage DNA adenine methylase, DNA primase, oxidoreductase, phage tail length tape measure protein, phage P2 protein GpE, tail protein, major tail tube protein, hypothetical proteins |
|  | GI-11 | 4708 | Peptide signal protein, signal peptide-containing protein, MerR family transcriptional regulator, hypothetical proteins |
|  | GI-12 | 4889 | 3-oxocyl-ACP synthase, DNA mismatch repair protein MutT, membrane protein, hypothetical proteins |
|  | GI-13 | 4339 | Toxin-antitoxin system, antitoxin component, membrane protein, DNA replication protein, hypothetical proteins |
|  | GI-14 | 6221 | Bacterial group 3 lg-like protein, conjugal transfer protein TraG, hypothetical proteins |
|  | GI-15 | 8530 | Pilus assembly protein, hypothetical proteins |
|  | GI-16 | 13475 | Initiator RepB protein, ParA family protein, fimbrial protein, hypothetical proteins |
|  | GI-17 | 17381 | Transcriptional regulator, sodium:proton exchanger, membrane protein, thioredoxin, Clp protease ClpC, heat-shock protein Hsp20, DNA-binding protein, pilus assembly protein, hypothetical proteins |
|  | GI-18 | 17791 | Antitoxin HicB, Cro/Cl family transcriptional regulator, transposase, MerR family transcriptional regulator, sodium:proton antiporter, lipoprotein signal peptidase, serine/threonine protein phosphatase, typtophan-tRNA ligase, hypothetical proteins |
|  | GI-19 | 5480 | Cytosinedeaminase, Fis family transcriptional regulator, cytosine permease, hypothetical proteins |
| **A362** | GI-1 | 7024 | Peptidase, M23 family protein, haloacid dehalogenase, hypothetical proteins |
|  | GI-2 | 6874 | Transporter, hypothetical proteins |
|  | GI-3 | 7251 | ATP-dependent Lon protease, transposase, hypothetical proteins |
|  | GI-4 | 14121 | Integrase, rifampin ADP-ribosylating transferase ARR-2, aminoglycoside adenylyltransferase, ethidium bromide resistance protein, transposase, extended-spectrum beta-lactamase PER-1, glutathione S-transferase, multidrug ABC transporter ATP-binding protein, transcriptional regulator, membrane protein, toxin-antitoxin system, antitoxin component, hypothetical proteins |
|  | GI-5 | 5518 | Bacteriophage protein, hypothetical proteins |
|  | GI-6 | 6767 | Catalase, resolvase, plasmid mobilization protein, hypothetical proteins |
|  | GI-7 | 7210 | PaaX family transcriptional regulator, carbonic anhydrase, phenylacetic acid degradation protein, alpha-ribazole phosphatase, hypothetical proteins |
|  | GI-8 | 10561 | Transcriptional regulator MerD, mercuric reductase, mercury transport protein MerC, MerR family transcriptional regulator, transposase, acetyltransferase, bacterial TniB protein, hypothetical proteins |
|  | GI-9 | 5416 |  |
|  | GI-10 | 10250 | Transposase, phosphoglucosamine mutase, dihydropteroate synthase, hypothetical proteins |
|  | GI-11 | 6526 | 3-oxoacyl-ACP synthase, GNAT family acetyltransferase, membrane protein, hypothetical proteins |
|  | GI-12 | 5431 | Darcynin 1, TetR family transcriptional regulator, hypothetical proteins |
|  | GI-13 | 4917 | Transposase, integrase, hypothetical proteins |
|  | GI-14 | 17594 | Phosphohydrolase, transposase, aminoglycoside phosphotransferase, transcriptional regulator, hypothetical proteins |
|  | GI-15 | 7913 | Hypothetical proteins |
|  | GI-16 | 25590 | RNA polymerase, transposase, integrase, conjugal transfer protein, diacylglycerol kinase, hypothetical proteins |
|  | GI-17 | 5458 | Transposase, hypothetical proteins |
|  | GI-18 | 5288 | Hypothetical proteins |
|  | GI-19 | 5838 | Ethidium bromide resistance protein, dihydropteroate synthase, nikA protein, XRE family transcriptional regulator, toxin HipA, hypothetical proteins |
|  | GI-20 | 8384 | Spore coat protein CotH, ParA family protein, initiator RepB protein, hypothetical proteins |
|  | GI-21 | 7163 | Aminoglycoside 3-N-acetyltransferase, tunicamycin resistance protein, transposase, hypothetical proteins |
| **NIPH973** | GI-1 | 4631 | Hypothetical proteins |
|  | GI-2 | 6937 | Hypothetical proteins |
|  | GI-3 | 6033 | Hypothetical proteins |
|  | GI-4 | 6265 | Hypothetical proteins |
|  | GI-5 | 5433 | DNA mismatch repair protein mutS, hypothetical proteins |
|  | GI-6 | 7910 | Hypothetical proteins |
|  | GI-7 | 7834 | Darcynin 2, Hypothetical proteins |
|  | GI-8 | 4483 | Hypothetical proteins |
|  | GI-9 | 34388 | Glutamate synthase large subunit-like protein, glutamine synthetase, type III, glycine cleavage system T protein, FolD1, formyltetrahydrofolate deformylase, formyltetrahydrofolate deformylase, nitrogen regulatory protein P-II, glycine cleavage system T protein, hypothetical proteins |
|  | GI-10 | 4716 | Hypothetical proteins |
