# Supplementary material for: Characterization of an Environmental Multidrug-Resistant Acinetobacter seifertii and Comparative Genomic Analysis Reveals Co-occurrence of Antimicrobial Resistance and Metal Tolerance Determinants
Source: Front Microbiol. 2019 Sep 18;10:2151. doi: 10.3389/fmicb.2019.02151 (PMC6759475; doi:10.3389/fmicb.2019.02151)
Supplement: Supplementary file 2 [file Table_2.DOCX]

**Supplementary Table 2 -** Phage-related sequences detected in *A. seifertii* strains.

| **Strains** | **Most Common Phage** | **Region Length (Kb)** | **GC %** | **Total Proteins** | **Completeness** |
| --- | --- | --- | --- | --- | --- |
| **SAb133** | - | - | - | - | - |
| **KCJK7915** | - | - | - | - | - |
| **KCJK1723** | PHAGE_Salmon_SEN34_NC_028699(22) | 45.1 | 38.25 | 49 | Intact |
| **1334_ABAU** | PHAGE_Acinet_Vb_AbaS_TRS1_NC_031098(3) | 11.4 | 38.06 | 13 | Incomplete |
| **MI421-133** | PHAGE_Synech_ACG_2014b_NC_027130(1)  PHAGE_Entero_phi92_NC_023693(2)  PHAGE_Prochl_P_SSM2_NC_006883(2) | 8.1  8.7  11.3 | 34.66  35.36  39.96 | 7  9  14 | Incomplete  Incomplete  Incomplete |
| **MI30-324** | PHAGE_Ralsto_RSS0_NC_019548(2)  PHAGE_Entero_Bp4_NC_024142(1) | 8.7  8.5 | 37.94  33.03 | 12  6 | Incomplete  Incomplete |
| **V1371** | PHAGE_Acinet_Bphi_B1251_NC_019541(28)  PHAGE_Acinet_vB_AbaS_TRS1_NC_031098(8) | 53.6  34 | 38.20  37.47 | 74  44 | Intact  Intact |
| **C917** | PHAGE_Bacter_Diva_NC_028788(2)  PHAGE_Entero_mEp235_NC_019708(4)  PHAGE_Acinet_vB_AbaS_TRS1_NC_031098(3) | 40.5  29.4  27.6 | 40.60  37.33  38.57 | 33  38  10 | Intact  Questionable  Incomplete |
| **A354** | PHAGE_Pelagi_HTVC010P_NC_020481(4) | 19.2 | 39.56 | 28 | Incomplete |
| **A360** | PHAGE_Salmon_SEN34_NC_028699(21)  PHAGE_Pseudo_phiCTX_NC_003278(15) | 51.7  40.7 | 39.69  39.50 | 61  50 | Intact  Intact |
| **A362** | PHAGE_Acinet_Bphi_B1251_NC_019541(13)  PHAGE_Acinet_vB_AbaS_TRS1_NC_031098(3)  PHAGE_Acinet_Bphi_B1251_NC_019541(9)  PHAGE_Salmon_SEN34_NC_028699(4) | 19.5  13.1  16.7  11.8 | 38.62  35.96  38.41  40.02 | 31  16  32  13 | Incomplete  Incomplete  Incomplete  Incomplete |
| **NIPH973** | PHAGE_Acinet_Bphi_B1251_NC_019541(33)  PHAGE_Pseudo_phiCTX_NC_003278(16) | 57.6  26.7 | 39.90  40.77 | 89  32 | Intact  Intact |
